# Capacity development and safety measures for health care workers exposed to COVID-19 in Bangladesh

**DOI:** 10.1186/s12913-021-07071-2

**Published:** 2021-10-11

**Authors:** Tapan Kumar Nath, Alak Paul, Dwaipayan Sikdar, Janardan Mahanta, Sujat Paul, Md Robed Amin, Shahanara Chowdhury, Md. Nur Hossain Bhuiyan, Md. Abdur Rob, Abdur Rahim, Md Khairul Islam, Md Mohiuddin Sharif, Kannan Navaneetham

**Affiliations:** 1 School of Environmental and Geographical Sciences, University of Nottingham Malaysia, Semenyih, Malaysia; 2 Department of Geography and Environmental Studies, University of Chittagong, Chittagong, Bangladesh; 3 Department of Biochemistry and Molecular Biology, University of Chittagong, Chittagong, Bangladesh; 4 Department of Statistics, University of Chittagong, Chittagong, Bangladesh; 5 Department of Medicine, Chittagong Medical College, Chittagong, Bangladesh; 6 Department of Medicine, Dhaka Medical College, Dhaka, Bangladesh; 7 Department of Obstetrics and Gynaecology, Chittagong Medical College, Chittagong, Bangladesh; 8 Department of Surgery, Chittagong Medical College, Chittagong, Bangladesh; 9 Senior Consultant (Medicine), Chattogram General Hospital, Chittagong, Bangladesh; 10 Junior Consultant (Medicine), Kuwait Bangladesh Friendship Government Hospital, Dhaka, Bangladesh; 11 Junior Consultant, Dhaka Medical College Hospital, Dhaka, Bangladesh; 12 Medical Officer, Dhaka Medical College Hospital, Dhaka, Bangladesh; 13 Department of Population Studies, University of Botswana, Gaborone, Botswana

**Keywords:** COVID-19, Health care workers, Personal protective equipment, infection prevention control, Bangladesh

## Abstract

**Background:**

The safety of health care workers (HCWs) in Bangladesh and the factors associated with getting COVID-19 have been infrequently studied. The aim of this study was to address this gap by assessing the capacity development and safety measures of HCWs in Bangladesh who have been exposed to COVID-19 and by identifying the factors associated with respondents’ self-reported participation in capacity development trainings and their safety practices.

**Methods:**

This cross-sectional study was based on an online survey of 811 HCWs working at 39 dedicated COVID-19 hospitals in Bangladesh. A pretested structured questionnaire consisting of questions related to respondents’ characteristics, capacity development trainings and safety measures was administered. Binary logistic regressions were run to assess the association between explanatory and dependent variables.

**Results:**

Among the respondents, 58.1% had been engaged for at least 2 months in COVID-19 care, with 56.5% of them attending capacity development training on the use of personal protective equipment (PPE), 44.1% attending training on hand hygiene, and 35% attending training on respiratory hygiene and cough etiquette. Only 18.1% reported having read COVID-19-related guidelines. Approximately 50% of the respondents claimed that there was an inadequate supply of PPE for hospitals and HCWs. Almost 60% of the respondents feared a high possibility of becoming COVID-19-positive. Compared to physicians, support staff [odds ratio (OR) 4.37, 95% confidence interval (CI) 2.25–8.51] and medical technologists (OR 8.77, 95% CI 3.14–24.47) were more exhausted from working in COVID-19 care. Respondents with longer duty rosters were more exhausted, and those who were still receiving infection prevention and control (IPC) trainings were less exhausted (OR 0.54, 95% CI 0.34–0.86). Those who read COVID-19 guidelines perceived a lower risk of being infected by COVID-19 (OR 0.44, 95% CI 0.29–0.67). Compared to the respondents who strongly agreed that hospitals had a sufficient supply of PPE, others who disagreed (OR 2.68, 95% CI 1.31–5.51) and strongly disagreed (OR 5.05, 95% CI 2.15–11.89) had a higher apprehension of infection by COVID-19.

**Conclusion:**

The findings indicated a need for necessary support, including continuous training, a reasonable duty roster, timely diagnosis of patients, and an adequate supply of quality PPE.

**Supplementary Information:**

The online version contains supplementary material available at 10.1186/s12913-021-07071-2.

## Background

SARS-CoV-2-induced coronavirus disease (COVID-19) caused an unfathomable disruption of social and personal lives at the global scale in mere weeks starting at the beginning of 2020 [1]. Originating from Wuhan, China, in December 2019 [2, 3], the highly contagious SARS-CoV-2 has spread to almost every corner of the world. As of September 6, 2021, the World Health Organization (WHO) has reported more than 221 million confirmed cases and about 4.6 million deaths globally [4]. The COVID-19 outbreak exposed health care workers (HCWs) at underprepared or unprepared health care facilities to a significant risk of being infected by this notoriously infectious virus due to having unavoidable close contact with patients during testing, isolation, and treatment, with their asymptomatic but infected attendants and with infected fellow HCWs [5–7]. The HCWs’ odds of being infected are augmented by repeated exposure to large numbers of infected patients, the stress of working intensively for extended hours without proper rest, hydration or nutrition coupled with the shortage of personal protective equipment (PPE) [8–10]. Reported COVID-19-confirmed cases in at least 152,888 HCWs globally indicated that HCWs were at disproportionate risk [11]. The situation clearly dictates the need for research on capacity development and measures to ensure the safety of HCWs in dedicated COVID-19 hospitals and the bearings of associated factors for determining measures to lessen the risks [9].

Bangladesh, which is one of the most densely populated countries in the world, reported its first COVID-19-positive case on March 8, 2020, which was speculated to be from expatriates who had returned from COVID-19-affected countries [12–14]. The total number of confirmed cases as of September 6, 2021, in Bangladesh was 1514,456 [15]. Similar to other countries, HCWs in Bangladesh are also at disproportionate risk of being infected by COVID-19. Bangladesh Medical Association (BMA) reports show that a total of 9403 health professionals, including 3111 physicians, 2274 nurses and 4018 other health staff, were infected, and 186 physicians had died of COVID-19 as of September 5 28, 2021 [16].

Health experts believe that HCWs in Bangladesh are exposed to a higher risk of COVID-19 infection due to the scarcity and low quality of PPE, lack of capacity development training on the use and disposal of safety gear, absence of effective infection control practices in hospitals, heavy workload with distress and fatigue, lack of quarantine facilities, and testing of patients for SARS-CoV-2 [17–20]. A report claimed that 24% of HCWs in Bangladesh had yet to receive PPE, and nearly 46% of them who received PPE were not satisfied with the quality [21]. A study conducted in a dedicated COVID-19 hospital in Bangladesh reported that a majority of HCWs (89%) did not receive adequate training on the use of PPE, and many of them (41%) reused PPE [22]. In an observational study among 500 HCWs in Bangladesh, researchers found that participants did not adhere to personal hygiene practices and reported a shortage of PPE in COVID-19 health care facilities [23]. Evidently, the safety of HCWs in Bangladesh and the factors associated with getting COVID-19 infection among them have been less studied. This cross-sectional study was an effort to address this gap, aiming to do the following:

i) assess capacity development and safety measures of HCWs in Bangladesh exposed to COVID-19, and
ii) identify the factors associated with respondents’ self-reported participation in capacity development trainings and their safety practices.

The findings of this study may assist health care policy-makers and practitioners in planning, implementing and following better occupational safety to lower the risks for HCWs working in COVID-19 care.

## Methods

### Study design and respondents

This cross-sectional study based on an online survey included 39 dedicated COVID-19 hospitals in Bangladesh. Two versions of the online self-administered questionnaire, in English and in *Bengali*, were created by using a Microsoft Office 365 form. Self-administered questionnaires can be distributed electronically via email or the internet, and the technique chosen depends on the amount and type of information desired, and the target sample size [24, 25]. The present self-administered survey was designed and conducted following the ACCADEMY group guide for clinicians [26]. Respondents consisted of HCWs, including physicians, nurses, medical technologists and support staff. The same questionnaire was provided except for variations in language to each of the respondents irrespective of their professional category. The questionnaire was shared through e-mail, Facebook messenger, WhatsApp, and online groups of HCWs working at dedicated COVID-19 hospitals followed by repeated requests for wider circulation among their colleagues to achieve a snowball sample of representative HCWs. To address possible duplicate answers, all authors followed strict guidelines about the questionnaire link and ensured the quality of the study through coordination. However, an electronic system was used to avoid double responses from similar links. The *Bengali* version was used for nurses and support staff who may face difficulties in providing responses on the English version. Participation in this survey was voluntary and anonymous, and respondents had the right to withdraw from the survey at any time. Before participating in the survey, prospective respondents had to answer a yes/no question to confirm their consent to participate voluntarily. After providing their online written consent, the respondents were requested to complete the questionnaire. The ethical review committee of Chittagong Medical College, Bangladesh approved this study (Memo No. CMC/PG/2020/97 dated 9 May 2020). The survey was carried out from 19 May 2020 to 19 September 2020. A total of 811 responses were obtained, which was satisfactory at the 95% confidence level with a ± 5% margin of error [27], thereby suggesting a suitable sample size (population size: 3000, sample size: 341) for its respective population. The response rate was approximately 32% of the total HCWs. The total number of HCWs in the 39 hospitals were 2502 including 952 physicians, 800 nurses, 201 medical technologists and 549 support staff [28].^1^ This report followed the STROBE statement (STROBE) on reporting cross-sectional studies in accordance with the Declaration of Helsinki for studies involving humans (Supplementary file 1).

### Measures

The questionnaire was prepared following the report of the World Health Organization [29] and the Ministry of Health and Family Welfare of Bangladesh [30] along with the authors’ prior research experience and mass media information, which was reviewed by two experts and underwent a pre-test with 40 respondents. To validate the questionnaire, experts were asked to assess its relevance for this study. Based on experts’ comments and pretest feedback, some questions were eliminated or rephrased for clarity. The final structured questionnaire consisted of 20 questions related to respondents’ characteristics, capacity development trainings, and safety measures (Supplementary file 2). Answering all questions was mandatory to justify a respondent as valid. On average, it took approximately 9 min to complete a questionnaire.

This study examined two major outcome measures. The first was capacity building of HCWs (two dependent variables, namely, participation in infection prevention and control (IPC)-related training and reading COVID-19-related guidelines) using gender, age, profession, professional experience, and experience with COVID-19 care as explanatory variables among the HCWs. The second outcome measure was safety measures of HCWs (three dependent variables namely, performed SARS-CoV-2 test, exhausted treating COVID-19 patients, and possibility of getting infected by COVID-19) using gender, age, profession, participation in IPC trainings, still receiving IPC trainings, read COVID-19 related guidelines, supply of PPE, all HCWs received PPE, duty roster, and exhausted treating COVID-19 patients as explanatory variables.

### Statistical analysis

After importing the responses from the online survey through Microsoft Excel, the data from both versions of the questionnaire were aggregated and cleaned by R programming and used for further analysis. Frequencies of single responses and multiple responses for different variables were estimated using the Statistical Package for the Social Sciences (SPSS for Windows, Version 16.0. Chicago, SPSS Inc.) and R programming (version 3.5.2), respectively. Binary logistic regressions were run to assess the association between explanatory and dependent variables. An odds ratio (OR) with a 95% confidence interval (CI) was used to assess the strength of the association, and a *P* value of less than 0.05 was considered significant. We also used adjusted odds ratios. Some explanatory variables with more than two categories were grouped into two categories, namely, “yes” and “no” (Supplementary file 3).

## Results

### Characteristics of the respondents

Most respondents were “male” (534 [65.8%]), and the majority of the respondents were in the “31–40 years” (449[55.4%]) and “up to 30 years” categories (275[33.9%]). Of the total respondents (811), 496 (61.2%) were physicians, 140 (17.3%) were support staff, 120 (14.8%) were nurses and 55 (6.8%) were medical technologists (Table 1), representing 52% physicians, 25.5% support staff, 15% nurses and 27.36% medical technologists of the total in each category involved in COVID-19 care during this study. Most respondents (467 [57.6%]) had “more than five years of professional experience”, followed by “1 – 3 years of professional experience” (218 [26.9%]). However, professional experience was not categorized based on profession.

**Table 1:** Background characteristics of respondents (*N* = 811)

Characteristics	n	Percent
Gender		
Male	534	65.8
Female	277	34.2
Age		
Up to 30	275	33.9
31–40	449	55.4
41–50	67	8.3
Above 50	20	2.5
Profession		
Physician	496	61.2
Nurse	120	14.8
Support staff	140	17.3
Medical technologist	55	6.8
Professional Experience		
Less than 1	82	10.1
1–3	218	26.9
4–5	44	5.4
More than 5	467	57.6
Experience with COVID-19 care (months)		
Less than 1	104	12.8
1–2	367	45.3
2–3	130	16.0
3–4	22	2.7
More than 4	188	23.2

A total of 188 (23.2%) respondents had “more than four months of experience”, while the majority (367 [45.3%]) had “1 – 2 months of experience” in caring for COVID-19 patients (Table 1).

### Capacity development trainings received by health care workers

We assumed that HCWs were not familiar with COVID-19 and had no experience with this kind of pandemic. Therefore, they received basic training from respective hospitals on managing highly contagious COVID-19 patients as frontline caregivers. Among the respondents, 458 (56.5%) attended training “on the use of personal protective equipment (PPE)”, 358 (44.1%) on “hand hygiene”, and 284 (35%) on “respiratory hygiene and cough etiquette” (Table 2). Surprisingly, one in every seven, i.e., 113 (13.9%), of them had not received any training. For 80% of the respondents, these trainings were fair to very useful. However, only 20% of the respondents continued to receive training.

**Table 2:** Capacity development trainings received by health care workers on COVID-19 and its quality (*N* = 811)

Training/quality indicators	n	Percent
Which of the following basic training did you attend? ^a^		
Hand hygiene	358	44.1
Respiratory hygiene and cough etiquette	284	35.0
Use of PPE	458	56.5
Decontaminate PPE/equipment/work surface/table/room, etc.	260	32.1
Safe handling of samples, cases and waste	109	13.4
Environmental decontamination and waste management	104	12.8
None of the above	113	13.9
Were these trainings useful?		
Very useful	493	60.8
Fairly useful	156	19.2
Not useful	13	1.6
Nothing new	36	4.4
Not applicable	113	13.9
Are you still receiving trainings?		
Yes	161	20.0
No	650	80.0
Have you read following COVID-19 related documents?^a^		
National Preparedness and Response Plan for COVID-19, Bangladesh	125	15.4
National Guideline for Health Care Providers on Infection Prevention and Control of COVID-19 pandemic in Health Care Settings	254	31.3
National Guidelines on the Clinical Management of Coronavirus Disease 2019 (Covid-19)	346	42.7
All of these documents	147	18.1
None of the above	263	32.4

Note: ^a^ multiple responses were allowed

The Ministry of Health and Family Welfare of Bangladesh published three important guidelines: i) National Preparedness and Response Plan for COVID-19, Bangladesh, ii) National Guideline for Health Care Providers on Infection Prevention and Control of COVID-19 pandemic in Health Care Settings, and iii) National Guidelines on the Clinical Management of Coronavirus Disease 2019 (COVID-19) to ensure the personal safety of HCWs and the proper management of COVID-19. Surprisingly, only 18.1% of the respondents had read all the guidelines, while one-third of them (32.4%) had not read any of these guidelines.

### Knowledge, perception and practice of safety measures among health care workers

Respondents’ perceptions of safety measures among HCWs are shown in Table 3. The majority of the respondents (54.2%) responded “agree” or “strongly agree” to the statement that hospitals had a sufficient supply of PPE. However, half (50.4%) of the respondents reported that they did not receive PPE, as substantiated by their claim that only (14.7%) of them changed PPE after attending a COVID-19 patient and 85.3% after every roster. Almost all of them reported practising proper hygiene (washing hands and showering) after returning home from hospitals. However, in contrast with recommendations, only one-third of them (31.9%) reported performing mandatory quarantines following their duty roster, while an alarming one-third of them (34.3%) reported going back to their family members after their rosters. For their own safety and others, it is important for all HCWs to undertake RT–PCR tests to detect SARS-CoV-2 before they begin duty in dedicated COVID-19 hospitals. However, only 3.8% of the respondents undertook this test at the start of their duty. Alarmingly, 59.7% of the respondents did not undertake an RT–PCR test even after their duty in COVID-19 hospitals.

**Table 3:** Knowledge, perception and practice of safety measures among health care workers (*N* = 811)

Safety measure indicators	n	Percent
Do you agree that your hospital has a sufficient supply of PPE?		
Strongly agree	56	6.9
Agree	384	47.3
Disagree	286	35.3
Strongly disagree	85	10.5
Do all health care staff receive PPE?		
Yes	402	49.6
No	409	50.4
When do you currently change your PPE?		
After attending a patient	119	14.7
After every roster	446	85.3
Where do you live now?		
At my residence, but in a separate room	274	33.8
At government/authority-designated accommodation	259	31.9
With family	278	34.3
What do you do immediately when you return from hospital duty to your accommodation/residence?		
Wash hands	49	6.0
Take a shower	84	10.4
Both	675	83.2
None	3	0.4
Duty roster		
Every day	270	33.3
7 days	322	39.7
10 days	184	22.7
14 days	35	4.3
After completion of your scheduled duty shift, do you undergo institutional quarantine for 14 days?		
Yes	286	35.3
No	397	49.0
Yes, but less than 14 days	128	15.7
When do you test for SARS-CoV-2?^a^		
At the start of your duty	31	3.8
When you are on duty	50	6.2
After completion of your duty	283	34.9
Do not test for SARS-CoV-2	484	59.7
If you are exhausted treating the COVID-19 patients, what are the reasons? ^a^		
Work overload	405	49.9
Panic	270	33.3
Lack of PPEs	275	33.9
Social disbelief	145	17.9
Not exhausted	215	26.5
What is the possibility of you getting COVID-19 infection?		
Low	76	9.3
Moderate	269	33.2
High	466	57.5
Many health care workers have already been infected with COVID-19. In your opinion, what might be the reasons?^a^		
Late diagnosis of COVID-19 patients	565	69.7
Longer duty hours	156	19.2
Sub-optimal adherence to prevention measures	217	26.8
Working in high-risk departments	236	29.1
Inadequate training on prevention measures	272	33.5
All of the above	384	47.3

Note: ^a^ multiple responses were allowed

Similar to other countries, HCWs in Bangladesh have also been exhausted, frightened, and tested positive for COVID-19. In this study, respondents reported several reasons for being exhausted when treating COVID-19 patients. While 50% of the respondents blamed ‘work overload’ as the cause for their exhaustion in treating COVID-19 patients, 33% identified ‘panic and lack of PPE’ as their reasons for exhaustion. Due to this fatigue, a majority (57.5%) of the respondents were highly afraid of becoming COVID-19-positive due to their service at dedicated COVID-19 hospitals. When asked about the probable reasons behind the perceived COVID-19 infections among HCWs, 69.7% of the respondents attributed late diagnosis of COVID-19 in patients as the prime reason, followed by inadequate training on prevention measures (33.5%), working in high-risk departments (29.1%), and suboptimal adherence to prevention measures (26.8%).

### Factors associated with capacity development training of health care workers

Among the HCWs, “medical technologists” were more interested in participating in IPC trainings (OR 5.77, 95% CI 2.21–15.06, *P* = 0.01) than physicians (Table 4). Those who had less experience with COVID-19 care were more likely to participate in IPC trainings. Compared to younger (up to 30 years) HCWs, others were reluctant to read COVID-19-related guidelines. For example, HCWs falling into the subgroups of “31–40 years” and “50 years and above” had 68% lower odds (OR 0.32, 95% CI 0.18–0.57, *P* = 0.001) and 82% lower odds (OR 0.18, 95% CI 0.05–064, *P* = 0.01) of reading COVID-19 guidelines than younger HCWs (Table 4). This indicated a greater willingness to acquire COVID-19-related knowledge among the younger HCWs. This observation was supported when compared to those having “less than 1 year” of professional experience, with those having “1–3 years” of professional experience showing less interest (OR 0.45, 95% CI 0.22–0.90, *P* = 0.04) in reading COVID-19 guidelines. Furthermore, those with “less than 1 month” of experience working with COVID-19 care were more motivated to study the guidelines than those with “3–4 months” of COVID-19 experience (OR 0.11, 95% CI 0.02–0.62, *P* = 0.01). Physicians were more interested in reading guidelines than other professional groups.

**Table 4:** Factors associated with capacity development of health care workers regarding COVID-19

Explanatory variable	Odds Ratio	95% CI	***P*** Value	Odds Ratio	95% CI	***P*** Value
Outcome: Capacity development	Participation in IPC trainings			Read COVID-19 related guidelines ^a^		
Gender						
Male	[1] Reference			[1] Reference		
Female	0.94	0.66–1.36	0.76	0.75	0.47–1.21	0.25
Age (years)						
Up to 30	[1] Reference			[1] Reference		
31–40	1.00	0.62–1.60	0.99	0.32	0.18–0.57	0.001
41–50	1.43	0.69–2.98	0.34	0.87	0.26–2.89	0.81
Above 50	1.10	0.38–3.16	0.87	0.18	0.05–0.64	0.01
Profession						
Physician	[1] Reference			[1] Reference		
Nurse	0.70	0.42–1.18	0.18	0.11	0.06–0.21	0.001
Support staff	0.71	0.43–1.16	0.17	0.08	0.04–0.14	0.001
Medical technologist	5.77	2.21–15.06	0.01	0.10	0.05–0.19	0.001
Professional experience (years)						
Less than 1	[1] Reference			[1] Reference		
1–3	0.55	0.31–0.98	0.04	0.45	0.22–0.90	0.02
4–5	0.50	0.22–1.16	0.11	0.37	0.12–1.13	0.08
More than 5	0.54	0.26–1.08	0.08	0.84	0.35–2.02	0.69
Experience with COVID-19 care (months)						
Less than 1	[1] Reference			[1] Reference		
1–2	0.66	0.23–1.93	0.45	0.29	0.05–1.57	0.15
2–3	0.69	0.23–2.06	0.50	0.20	0.04–1.09	0.06
3–4	0.46	0.14–1.47	0.19	0.11	0.02–0.62	0.01
More than 4	0.81	0.28–2.39	0.70	0.26	0.05–1.41	0.12

Note: ^a^ Please see Supplementary file 2 for categories of responses for logistic regressions; *IPC* Infection prevention and control

### Factors associated with safety measures of health care workers

Table 5 shows that compared to younger HCWs, those “above 50 years” were quite unlikely to undergo RT–PCR (OR 0.16, 95% CI 0.03–0.74, *P* = 0.02). Among professional groups, support staff were more willing to perform RT–PCR tests (OR 1.95, 95% CI 1.17–3.28, *P* = 0.01). Those who attended IPC trainings (OR 1.46, 95% CI 1.02–2.07, *P* = 0.04), had read COVID-19 guidelines (OR 3.31, 95% CI 2.14–5.12, *P* = 0.01), responded as having received PPE (OR 1.59, 95% CI 1.07–2.37, *P* = 0.02), and had duty rosters of 7 days (OR 1.69, 95% CI 1.13–2.54, *P* = 0.01) and 10 days (OR 2.39, 95% CI 1.50–3.81), *P* = 0.01) were more likely to go for RT–PCR tests. Respondents who strongly disagreed that hospitals had a sufficient supply of PPE had 74% lower odds of performing RT–PCR tests (OR 0.26, 95% CI 0.11–0.61, *P* = 0.01).

**Table 5:** Factors associated with safety measures of health care workers

Explanatory variable	Odds ratio	95% CI	***P*** Value	Odds ratio	95% CI	***P*** Value	Odds Ratio	95% CI	***P*** value
Outcome: Safety measures	Performed SARS-CoV-2 test ^a^			Exhausted treating COVID-19 patients ^a^			Possibility of getting infected by COVID-19 ^a^		
Gender									
Male	[1] Reference			[1] Reference			[1] Reference		
Female	0.86	0.60–1.24	0.43	1.30	0.83–2.03	0.25	0.94	0.66–1.35	0.74
Age (years)									
Up to 30	[1] Reference			[1] Reference			[1] Reference		
31–40	1.04	0.73–1.47	0.84	0.87	0.58–1.31	0.51	1.24	0.89–1.74	0.20
41–50	0.66	0.36–1.20	0.17	0.67	0.35–1.30	0.24	0.67	0.37–1.22	0.19
Above 50	0.16	0.03–0.74	0.02	0.28	0.10–0.79	0.02	0.67	0.24–1.84	0.44
Profession									
Physician	[1] Reference			[1] Reference			[1] Reference		
Nurse	0.89	0.50–1.56	0.68	0.76	0.41–1.41	0.38	1.75	1.00–3.04	0.05
Support staff	1.95	1.17–3.28	0.01	4.37	2.25–8.51	0.001	1.17	0.71–1.93	0.55
M. technologists	0.98	0.46–2.08	0.95	8.77	3.14–24.47	0.001	1.61	0.77–3.37	0.21
Participation in IPC trainings									
No	[1] Reference			[1] Reference			[1] Reference		
Yes	1.46	1.02–2.07	0.04	1.50	0.98–2.29	0.06	0.95	0.67–1.35	0.77
Still receiving IPC trainings									
No	[1] Reference			[1] Reference			[1] Reference		
Yes	0.98	0.65–1.49	0.94	0.54	0.34–0.86	0.001	1.22	0.81–1.83	0.35
Read COVID-19 related guidelines									
No	[1] Reference			[1] Reference			[1] Reference		
Yes	3.31	2.14–5.12	0.01	0.65	0.40–1.07	0.09	0.44	0.29–0.67	0.001
Hospitals had a sufficient supply of PPE									
Strongly agree	[1] Reference			[1] Reference			[1] Reference		
Agee	0.45	0.24–0.86	0.02	1.49	0.77–2.92	0.24	1.42	0.76–2.66	0.28
Disagree	0.55	0.27–1.13	0.10	2.81	1.28–6.17	0.01	2.68	1.31–5.51	0.01
Strongly disagree	0.26	0.11–0.61	0.01	7.68	2.59–22.73	0.001	5.05	2.15–11.89	0.001
All HCWs received PPEs									
No	[1] Reference			[1] Reference			[1] Reference		
Yes	1.59	1.07–2.37	0.02	0.67	0.42–1.05	0.08	1.34	0.90–1.99	0.15
Duty roster									
Every day				[1] Reference			[1] Reference		
7 days	1.69	1.13–2.54	0.01	2.45	1.55–3.85	0.001	1.11	0.75–1.65	0.59
10 days	2.39	1.50–3.81	0.01	3.05	1.81–5.15	0.001	1.45	0.92–2.30	0.11
14 days	1.36	0.62–3.02	0.45	2.56	0.94–6.96	0.07	1.02	0.47–2.18	0.97
HCWs’ exhaustion treating COVID-19									
No	[1] Reference						[1] Reference		
Yes with some reasons	1.31	0.89–1.92	0.18				1.98	1.38–2.86	0.001

^a^Please see Supplementary file 2 for categories of responses for logistic regressions; *IPC* Infection prevention and control

Compared to the younger HCWs, the oldest group of HCWs “above 50 years” (OR 0.28, 95% CI 0.10–0.79, *P* = 0.02) was less likely to become exhausted in treating COVID-19 patients, probably due to their long professional experience. However, in reference to physicians, “medical technologists” (OR 8.77, 95% CI 3.13–24.47, *P* = 0.001) and “support staff” (OR 4.37, 95% CI 2.25–8.51, *P* = 0.001) were more exhausted. HCWs who were still receiving IPC-related training had 46% lower odds of being exhausted when treating COVID-19 patients than those who received no training (OR 0.54, 95% CI 0.34–0.86, *P* = 0.001). Respondents who disagreed (OR 2.81, 95% CI 1.28–6.17, *P* = 0.01) and strongly disagreed (OR 7.68, 95% CI 2.59–22.73, *P* = 0.001) that hospitals had a sufficient supply of PPE were more exhausted in treating COVID-19 patients than those who strongly agreed that they had a sufficient PPE supply.

The duty roster was also associated with HCW exhaustion. HCWs with duty rosters of 7 days (OR 2.45, 95% CI 1.55–3.85, *P* = 0.001) and 10 days (OR 3.05, 95% CI 1.81–5.15, *P* = 0.001) were more exhausted than those who were in a daily roster.

Nurses (OR 1.75, 95% CI 1.00–3.04, *P* = 0.05), support staff, and medical technologists perceived a higher risk of being infected by COVID-19 than physicians (Table 5). Those who read COVID-19-related guidelines had a 56% lower odds risk of being infected by COVID-19 (OR 0.44, 95% CI 0.29–0.67, *P* = 0.001) than those who did not read these guidelines. Compared to those who strongly agreed that hospitals had a sufficient supply of PPE, higher fears of becoming COVID-19-positive prevailed among HCWs who ‘disagreed’ (OR 2.68, 95% CI 1.31–5.51, *P* = 0.01) and ‘strongly disagreed’ (OR 5.05, 95% CI 2.15–11.89, *P* = 0.001) about the sufficiency of the PPE supply. HCWs who were exhausted for several reasons had a higher possibility of being infected by COVID-19 than those who were not exhausted (OR 1.98, 95% CI 1.38–2.86, *P* = 0.001).

## Discussion

Research on the safety measures and practices of HCWs exposed to COVID-19 is still in its infancy [31]. The COVID-19 pandemic has brought great challenges to health care systems, and many health care providers from other departments who have limited clinical experiences in infectious intensive care need capacity development in terms of training, education, and improved communication to handle the outbreak of an infectious disease [32, 33]. In this study, physicians participated most often among HCWs, followed by support staff and nurses, indicating the increased willingness of physicians to participate in such surveys possibly due to their past exposure, while among nurses, their reluctance may be due to their lack of experience in participating in such research. At the onset of COVID-19, the Government of Bangladesh prepared a number of hospitals in the capital city, Dhaka, for the treatment of COVID-19 patients and deployed HCWs. As transmission spread, new hospitals were dedicated to the treatment of COVID-19. Accordingly, HCWs had a varying duration of exposure to COVID-19 cases or contacts, as reflected in the present findings. We assumed that HCWs in Bangladesh probably had no previous experience dealing with global pandemics such as COVID-19. Therefore, HCWs who were delegated to dedicated COVID-19 hospitals were required to develop their capacity by joining in trainings on IPC and acquiring knowledge by reading national and international guidelines to impart better health care while keeping themselves safe. The findings of this study show that a substantial portion of respondents have not received hands-on training and had even discontinued (80% respondents) receiving training. Moreover, only 18% of the respondents had educated themselves on the three important guidelines relevant to COVID-19 care, which indicated the lack of preparedness as professionals to handle pandemics such as COVID-19. It was suggested that HCWs should be trained on IPC, proper ways of wearing and taking off PPE, and adhering to standard recommendations in clinical settings with a high risk of exposure to reduce the risk of infection [34, 35]. Acquiring knowledge and continuous training on critical care can increase the professional knowledge of HCWs, improve their practical skills, and help them deal with public health emergencies [33, 36]. Health care providers are expected to remain in continuous training and remain up-to-date on essential guidelines to improve knowledge on infection prevention and control [37].

In addition to capacity development, it is important to ensure a safe working environment and access to sufficient and reliable PPE for HCWs [5, 37, 38]. The findings of this study show that due to the lack of availability of PPE, only 50% of the respondents received PPE, and their PPE was inadequately supplied, as only 85.3% of them changed their PPE after every roster. The shortage of PPE is a global issue, and HCWs are often forced to share PPE and reuse disposable PPE [39, 40]. To protect HCWs, it is essential to ensure the availability of adequate PPE for all HCWs, and governments need to expedite the procurement and devise a strategy for the use of available PPE [31]. Reuse of PPE may increase the risk of infection, and it was reported that those who reused their medical gowns had a twofold greater change of testing positive for COVID-19 than those who had not reused them [41]. Like many countries, Bangladesh is experiencing the second and third waves of COVID-19 infection. As such, the government needs to take measures for continuous trainings on IPC and the procurement of sufficient PPE.

After their duty roster, contrary to the guidelines, only 35% of respondents went under formal quarantine, and many (34%) were living with family members. HCWs exposed to COVID-19 are a substantial source of disease transmission to others and their family members, which necessitates ensuring their strict adherence to maintaining a quarantine protocol [1, 42]. Hospitals are becoming hotspots for disease transmission; therefore, HCWs should be screened for COVID-19 every week to protect non-COVID-19 patients from asymptomatic patients [43]. Actively testing HCWs for SARS-CoV-2 will be key in swiftly identifying, isolating, supporting, and reintroducing infected HCWs following recovery [39]. HCWs need to be tested for SARS-CoV-2 prior to starting their next duty cycle at COVID-19 hospitals to check the spread of the disease. Only 40% of the respondents underwent the SARS-CoV-2 test after their roster, indicating a high chance of COVID-19 transmission through HCWs if they were not properly quarantined. A lack of testing of health care workers during their roster can transmit the disease to other HCWs working in the same hospitals.

Our survey respondents were exhausted treating COVID-19 patients due to longer duty rosters (50% respondents), panic situations (33%) and a lack of PPE (34%). The present study shows that those who had 7- or 10-day duty rosters were more exhausted than those who had daily duty rosters. This implied that HCWs in daily rosters could return to their family after their hospital duty and spend time with family members and thus were not as exhausted working in dedicated COVID-19 hospitals. Conversely, HCWs in other rosters had to stay at designated accommodations, and they were probably emotionally exhausted. Nearly 60% of the respondents perceived a high possibility of contracting COVID-19, and 70% opined that the late diagnosis of COVID-19 in patients was the principal reason for the presence of already infected HCWs in Bangladesh. Globally, the present situation has affected the mental health of many HCWs, which may reduce their resilience in the face of future waves of COVID-19 [44]. Researchers have reported that long duty hours under significant work pressure with often inadequate resources place HCWs under serious danger of COVID-19 infection [1, 7, 38]. The rate of infection can be lowered with the use of PPE combined with proper training on infection control [45]. It has been reported that along with the quality of PPE, patients who conceal their symptoms of COVID-19 are also responsible for HCWs’ infection [46].

### Limitations of this study

This study had a number of limitations. Even though we repeatedly requested that the targeted respondents to complete the survey, the overall participation rate was not very high, especially among females. Physical interviews, if possible, with different groups of HCWs would be useful to increase the participation rate. However, our assumption was that those who had participated in the survey were randomly selected; hence, the characteristics between those who responded and those who did not were the same. Therefore, we expect that the low response rate would not alter the results or the conclusions derived from them. HCWs engaged with COVID-19 care suffer from various types of psychological distress. In this study, we had no questions related to mental health issues among HCWs exposed to COVID-19. We considered the professional experience of HCWs, but there was no distinction based on profession, with similar findings regarding experience with COVID-19 care.

## Conclusions

Health care workers are at the frontline in response to the COVID-19 outbreak, which makes them vulnerable to a higher risk of infection. This study identified factors such as inadequate training on IPC, lack of personal knowledge acquisition on COVID-19 guidelines, limited access to PPE, long duty rosters, late diagnosis of COVID-19 disease among patients, and lack of regular screening for SARS-CoV-2 as the most likely reasons for their disproportionate risk of becoming COVID-19-positive. Thus, the results of this study may be helpful to policy-makers in Bangladesh to adopt a proper strategy to minimize the loss of valuable lives of HCWs and ensure their service to COVID-19 patients in the country.

## Supplementary Information

**Additional file 1.**

**Additional file 2.**

**Additional file 3.**

## Data Availability

All data generated or analysed during this study are included in this article [and its supplementary information files].

## Footnotes

1 At the beginning of COVID-19 pandemic in Bangladesh, the government initially allocated several medical units in 39 hospitals only for COVID-19 treatment. As of 29 May 2020, the total number of HCWs were 2502 [28]. However, the government recruited new HCWs when the COVID-19 situation was worsened.
