# Supplementary material for: Capacity development and safety measures for health care workers exposed to COVID-19 in Bangladesh
Source: BMC Health Serv Res. 2021 Oct 11;21:1079. doi: 10.1186/s12913-021-07071-2 (PMC8504780; doi:10.1186/s12913-021-07071-2)
Supplement: Supplementary file 2 — Additional file 2. [file 12913_2021_7071_MOESM2_ESM.docx]

**S2 Table. Questionnaire used for online survey in Bangladesh.**

| 1. **Respondents’ characteristics** | |
| --- | --- |
| Q1. What is your gender? | Male, Female |
| Q2. What is your age? (years) | <30, 31-39, 40-49, >50 |
| Q3. What is your profession? | Physician, Nurse, Medical technologists & support staff |
| Q4. How long have you been with above profession? (years) | <1, 1-3, 4-5, >5 |
| Q5. How long have you been involved with COVID-19 treatment? (months) | <1, 1-2, 2-3, 3-4, >4 |
| 1. **Capacity development trainings and COVID-19 related guidelines** | |
| Q6. Which of the following basic training did you attend? Multiple answer was allowed. | Hand hygiene (soap-water/ hand sanitizer/70% ethanol)  Respiratory hygiene and cough etiquette (cover cough-sneeze)  Personal protective equipment (PPE) use  Decontaminate PPE/equipment/work surface/table/room etc.  Safe handling of sample, case and waste  Environmental decontamination and waste management  None of above |
| Q7. Are these training useful? | Very useful, Fairly useful, Not useful, Nothing new, Not applicable |
| Q8. Do you still receive trainings? | Yes, No |
| Q9. Have you read following documents? Multiple answer was allowed. | National Preparedness and Response Plan for COVID-19, Bangladesh  National Guideline for Health Care Provider On Infection Prevention and Control of COVID-19 pandemic in Healthcare Setting  National Guidelines on Clinical Management of Coronavirus Disease 2019 (Covid-19  All documents  None of above |
| 1. **Safety measures related questions** | |
| Q10. Do you agree that your hospital has enough supply of PPEs? | 1= Strongly agree, 2= Agree, 3= Disagree, 4= Strongly disagree |
| Q11. Do all healthcare staff receive PPE? | Yes, No |
| Q12. When do you change a PPE now-a-days? | After attending a patient, After every shift (roster) |
| Q13. Where do you live now? | At my residence, but in a separate room; At government/authority designated accommodation; With family |
| Q14. What do you do immediately when you return from hospital duty to your accommodation/residence? | Wash hands; Have shower; Both; None |
| Q15. What is you roster duty now-a-days? | Every day, 7 days, 10 days, 14 days |
| Q16. After completion of your scheduled duty shift, do you undergo Institutional quarantine for 14 days? | Yes; No; Yes, but less than 14 days |
| Q17. When do you test SAR-CoV-2? Multiple answer was allowed. | At the start of your duty; When you are on duty; After completion of duty; Do not test SAR-CoV-2 |
| Q18. If you are exhausted treating the COVID-19 patients, what are the reasons? Multiple answer was allowed. | Due to overload of work; Due to panic; Lack of PPE; Social disbelief; Not exhausted |
| Q19. How much possibility of you getting COVID-19 infection? | 1= Low, 2= Moderate, 3= High |
| Q20. Many healthcare workers already infected by COVID-19. In your opinion, what might be the reasons? Multiple answer was allowed. | Late diagnosis of COVID-19 patients; Longer duty hours; Sub-optimal adherence to prevention measures; Working in high risk department; Inadequate training on prevention measures; All of above |
