# Supplementary material for: Capacity development and safety measures for health care workers exposed to COVID-19 in Bangladesh
Source: BMC Health Serv Res. 2021 Oct 11;21:1079. doi: 10.1186/s12913-021-07071-2 (PMC8504780; doi:10.1186/s12913-021-07071-2)
Supplement: Supplementary file 3 — Additional file 3. [file 12913_2021_7071_MOESM3_ESM.doc]

**Supplementary Table 3: Categories of responses for logistic regression**

| Variable | Category | Binary Category for Logistic Regression |
| --- | --- | --- |
| Participation in IPC trainings | Hand hygiene | Yes: Others/ Taking any training (1) |
|  | Respiratory hygiene and cough etiquette |  |
|  | Personal protective equipment (PPE) use |  |
|  | Decontaminate PPE/equipment/work surface/table/room etc. |  |
|  |  |  |
|  | Safe handling of sample, case and waste |  |
|  | Environmental decontamination and waste management |  |
|  | None of above | No (0) |
|  | | |
| Read COVID-19 related guidelines | National Preparedness and Response Plan for COVID-19, Bangladesh | Yes: Others/ Read any guideline (1) |
|  | National Guideline for Health Care |  |
|  | Provider On Infection Prevention and Control of COVID-19 pandemic in Healthcare Setting |  |
|  | National Guidelines on Clinical |  |
|  | Management of Coronavirus Disease 2019 (Covid-19 |  |
|  | All documents |  |
|  | None of above | No (0) |
|  | | |
| Performing SAR-CoV-2 tests | At the start of your duty | Yes: Others/ Doing any test (1) |
|  | When you are on duty |  |
|  | After completion of duty |  |
|  | Do not test SAR-CoV-2 | No (0) |
|  | | |
| Exhaustion treating COVID-19 patients | Due to overload of work | Yes: Others/ Have some reasons (1) |
|  | Due to panic |  |
|  | Lack of PPEs |  |
|  | Social disbelief |  |
|  | Not exhausted | No (0) |
|  | | |
| Possibility of getting infected by COVID-19 | High | Yes: High (1) |
|  | Low | No: Others (0) |
|  | Moderate |  |
